# Numerical Simulation of Pipeline Deformation Caused by Rockfall Impact

**DOI:** 10.1155/2014/161898

**Published:** 2014-05-13

**Authors:** Jie Zhang, Zheng Liang, Chuanjun Han

**Affiliations:** School of Mechatronic Engineering, Southwest Petroleum University, Chengdu 610500, China

## Abstract

Rockfall impact is one of the fatal hazards in pipeline transportation of oil and gas. The deformation of oil and gas pipeline caused by rockfall impact was investigated using the finite element method in this paper. Pipeline deformations under radial impact, longitudinal inclined impact, transverse inclined impact, and lateral eccentric impact of spherical and cube rockfalls were discussed, respectively. The effects of impact angle and eccentricity on the plastic strain of pipeline were analyzed. The results show that the crater depth on pipeline caused by spherical rockfall impact is deeper than by cube rockfall impact with the same volume. In the inclined impact condition, the maximum plastic strain of crater caused by spherical rockfall impact appears when incidence angle *α* is 45°. The pipeline is prone to rupture under the cube rockfall impact when *α* is small. The plastic strain distribution of impact crater is more uneven with the increasing of impact angle. In the eccentric impact condition, plastic strain zone of pipeline decreases with the increasing of eccentricity *k*.

## 1. Introduction

**Figure 1:**
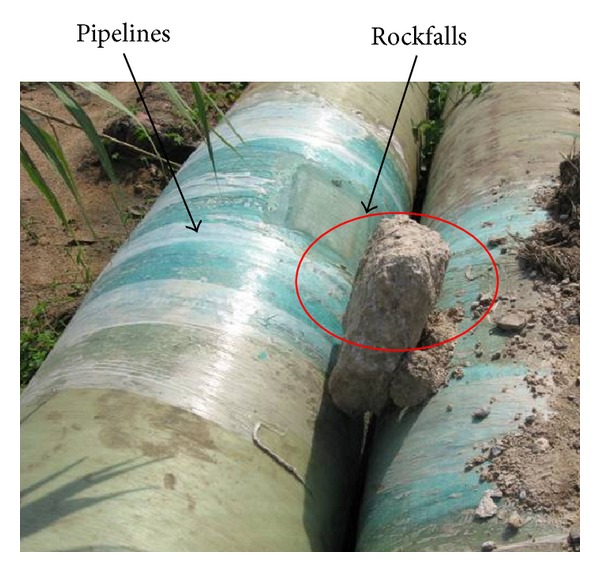
Rockfalls impact pipelines.

Long-distance oil and gas pipelines are usually laid underground, while the pipelines are laid on the ground when they go across valleys, rivers, swamps, deserts, permafrost, and other regions [1]. The pipelines on the ground have little effect on the soil environment, and the replacement or maintenance of pipelines on the ground is much more convenient. However, pipelines are vulnerable to external shock loads and wind loads because they are exposed at the ground's surface (see Figure 1). For instance, in April 2005, a pipeline's impact deformation caused by dangerous rockfall took place in Chongqing [2]. Since the pipelines are put into production in 2002, some incidents happened that rockfall impacted the pipelines along Lanzhou—Chengdu—Chongqing Products Pipeline. The rockfall impact has become one of the most serious geological disasters to endanger the safety of the pipeline [3]. The rockfall impact is subject to cause pipeline partial concave or rupture, which easily leads to leakage accident of oil or gas. Therefore, research on pipeline deformation caused by rockfall impact is important for the design, protection, and safety assessment of pipelines.

Currently, researchers often use two kinds of simplified models to research the rockfall impact problems. One is to simplify the rockfall to spherical structure. He et al. [4] and Wang et al. [5] studied the dynamic response problem of the shed-tunnel structure under the rockfall impact. Another is to simplify the rockfall to cube structure. As such, Xiong et al. [2] and Deng et al. [6] analyzed the dynamic response of buried pipes when the cube rockfall impacted the ground by numerical simulation. Based on the actual working conditions of pipeline under rockfall impact, this paper simulated the impact processes of pipelines under spherical and cube rockfall impacting. Also the deformation and plastic strain of pipeline were discussed.

## 2. Elastic-Plastic Failure Criterion

If oil and gas pipelines are elastic-plastic materials, high strain and strain rate effect should be taken into consideration as these may be significant during rockfall impact condition [7]. Currently, the material constitutive models used in impact problem include Cowper-Symonds model, Johnson-Cook model, and Zerilli-Armstrong model. It not only considers the role of strain and strain rate, but also takes the material softening caused by temperature changes produced by plastic expansion into consideration in the latter two models. According to the velocity range of rockfall impact of oil and gas pipeline, the paper chose the Cowper-Symonds model as the constitutive model of pipeline, which ignores the influence of temperature on material properties. Therefore, the model is applicable to analysis of high strain rate impact. The following expression is the dynamic yield stress of the model [8]:

(1) $$\begin{array}{c} \sigma_{y}=\left[1+{\left(\frac{\dot{\varepsilon }}{C}\right)}^{1/P}\right]\left(\sigma_{0}+\beta E_{P}\varepsilon^{\text{eff}}\right), \end{array}$$

where $\dot{\varepsilon }$ is the strain rate, *σ*_0_ is the initial yield stress, *ε* ^eff^ is the equivalent plastic strain, *β* is the hardening parameter, *E*_*p*_ is the plastic hardening modulus, and *C* and *P* are the coefficients of strain rate, respectively. The plastic reinforcement modulus is

(2) $$\begin{array}{c} E_{P}=\frac{EE_{\tan}}{E+E_{\tan}}, \end{array}$$

where *E* is the elasticity modulus and *E*_tan_ is the tangent modulus.

As the impact is a highly nonlinear problem [9], the failure destruction is defined with the rupture strain failure criteria, which means that when the plastic strain of finite element mesh is bigger than the maximum effective plastic strain of the material, the pipeline will have a fracture failure.

## 3. The Finite Element Model

### 3.1. Examples Validation

The experimental model of a flat projectile transverse impact pipeline was established in [10], and valuable results were obtained with system test. The same impact model was established by nonlinear finite element program ADINA. The explicit integral algorithm was used to simulate the impacting process. The length of the flat nose is 30 mm, the diameter is 15 mm, and the quality is 41 g. The pipeline's thickness is 3.46 mm, and the outside diameter is 115 mm. The Cowper-Symonds material model and the fracture strain failure criteria were used. Penalty contact algorithm was selected; the friction coefficient between rock and pipelines is 0.3 [2].

**Figure 2:**
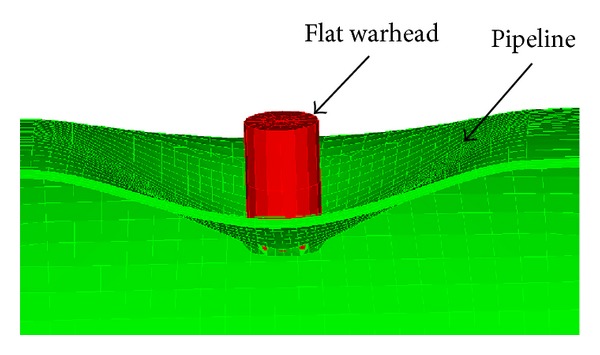
The simulation results of example.

Mesh size has a great effect on the simulation results. Many attempts have been made to find the most suitable mesh size. When the mesh size of the pipeline is 2.5 mm, the critical state of the pipeline rupture is obtained as shown in Figure 2. A large pit and partial rupture appear on the impact zone. The critical rupture velocity *V* is 186 m/s, while the experimental test velocity is 181 m/s, so the error is 2.76%. The critical rupture internal energy *E* (*E* = 1/2*mV* ^2^) obtained by simulation is 709.2 J, which is bigger than the experimental value 671.6 J; the error is 6%. Obviously, the numerical simulation results are in good agreement with the test dates, which proved that the finite element model and mesh size are reliable.

### 3.2. The Finite Element Model

In the calculation, the spherical and cube rockfalls have the same volume and homogeneous material [11]. Spherical rockfall's radius is 100 mm, the cube rockfall is regular hexahedron, side length is 160 mm, the density of rockfall is 2700 kg/m^3^, the elasticity modulus is 55.8 GPa, and Poisson ratio is 0.25. It is assumed that the material of pipeline is the ideal elastic-plastic material, the outer diameter is 599 mm, the thickness is 10 mm, the elastic modulus is 210 GPa, the density is 7800 kg/m^3^, Poisson ratio is 0.3, the material's limit fracture strain is 0.74 [9], the limit yield stress is 443 Mpa, and elongation is 0.37 [9]. In this example, the internal pressure of the pipeline is 5 MPa, and span length is 2.6 m.

**Figure 3:**
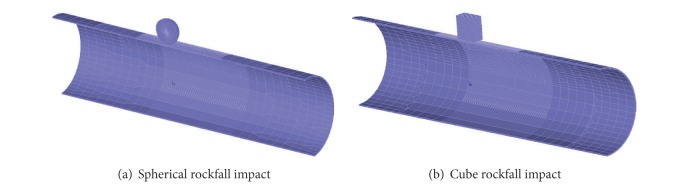
Three-dimensional finite element models.

Considering symmetry of the structure model, the 1/2 entire model was selected to analyze. The calculation model after meshing is shown in Figure 3. The mesh size of pipeline is 2.5 mm, which is the same as the example in Figure 2. Symmetry planes of the model were separately imposed on symmetry constraint; one end of the pipeline is fixed, and the other one is constrained in all directions except the axial direction. Then, the contact relationship between rockfall and pipeline with a penalty function was established, gravity load was applied to the whole model, and the acceleration due to gravity is 9.8 m/s^2^.

## 4. Results and Discussions

### 4.1. Deformation Analysis under Normal Impact

**Figure 4:**
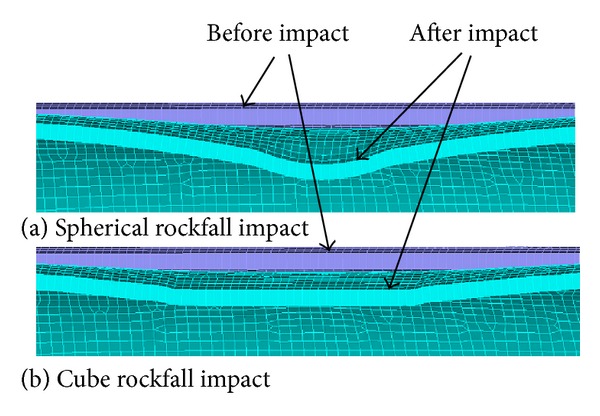
Pipeline deformation before and after impact.

The pipeline deformation caused by the rockfall impact along the normal direction is shown in Figure 4. Spherical rock causes a spherical cone concave at the bottom of the indentation, while the cube rock causes a rectangular concave. So, the shape of impact crater is related to the shape of rockfall. In addition to the impact contact place, plastic deformation is observed at near site. The further the observation site from the contact place, the smaller the deformation.

**Figure 5:**
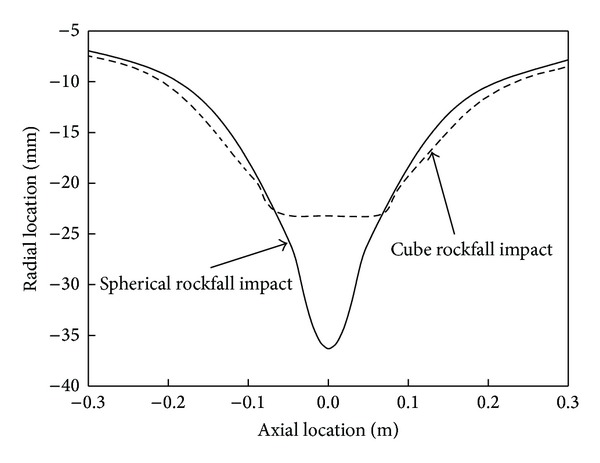
Shape caves of the two impact craters in the longitudinal plane.

The section view of craters impacted by different rocks in the longitudinal plane is shown in Figure 5. With the same volume of rockfalls, the impact crater caused by spherical rock is deeper. There is a small difference between the no-impact zones of the two curves.

**Figure 6:**
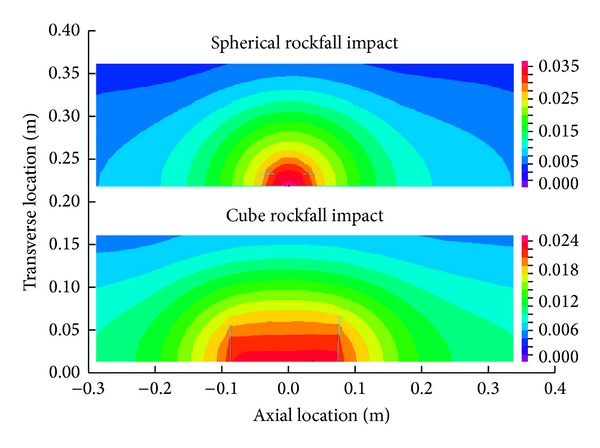
Deformation of the two impact craters.

As shown in Figure 6, the deformations of pipelines are radial distribution. The farther away from the impact zone, the smaller the deformation. Spherical rockfall can make an oval concave, while the cube rockfall makes a rectangle concave.

**Figure 7:**
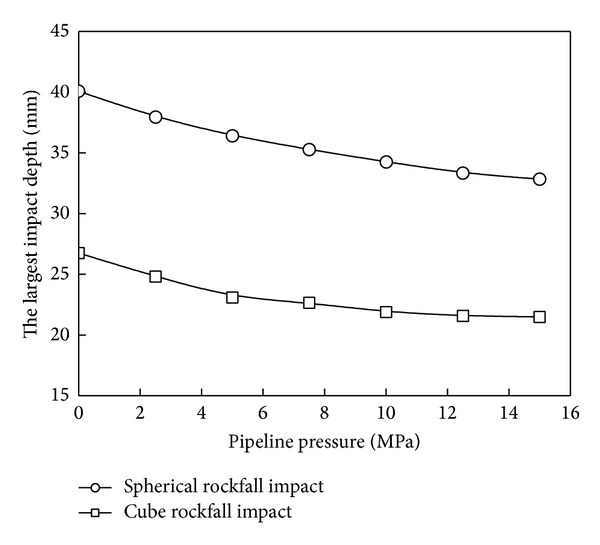
Effects of pressure on impact crater depth.

Internal pressure has a great effect on the shock stiffness of the pipeline. The largest impact crater depth values under different pipeline pressure are shown in Figure 7. As the internal pressure increases gradually, the largest impact crater depth decreases. When the pressure is 15 MPa, the biggest impact depths are 82.0% and 80.2%, respectively, under spherical and cube rockfalls. So, it is necessary to take internal pressure into consideration when studying the pipeline deformation caused by rockfall impact.

**Figure 8:**
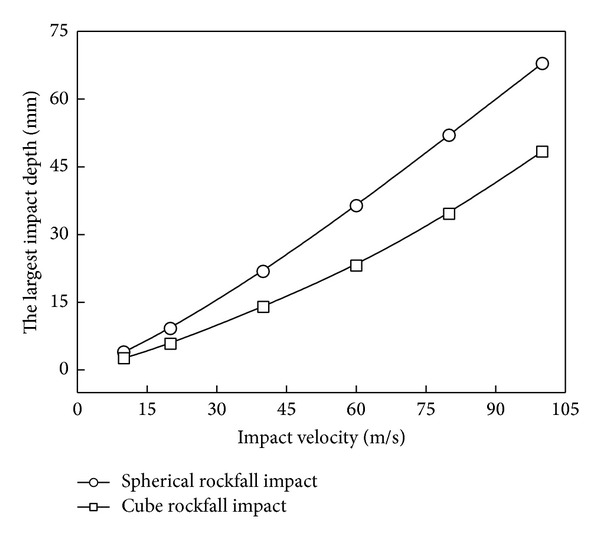
Effects of impact velocity on impact depth.

Figure 8 shows the largest impact crater depth under different impact velocity. With the increasing of impact velocity, both impact depth values increase gradually. The smaller the impact velocity, the closer the two impact depths. With the same velocity change range, the change of impact depth of spherical rockfall is bigger than cube rockfall. It means that spherical rockfall impact crater is more sensitive to impact velocity.

**Figure 9:**
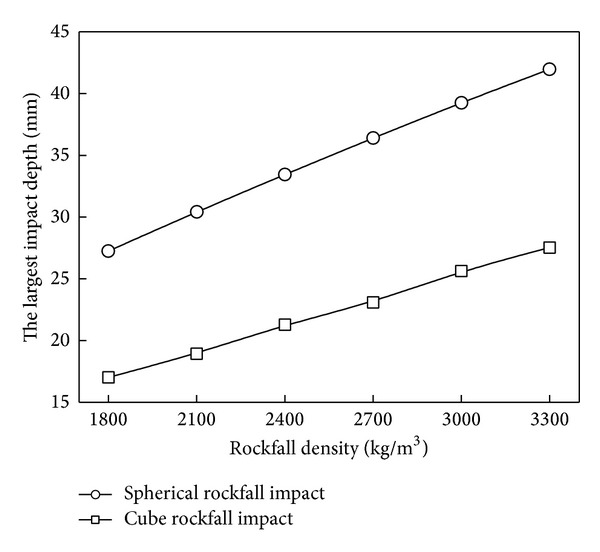
Effect of rockfall density on impact depth.

The density of rock affects its quality and then affects the impulse. The largest impact crater depth values under different rock density are shown in Figure 9. The greater rockfall density, the more serious pipelines' impact deformation. So, the more effective protective measures are needed for the rockfall with large density.

### 4.2. Deformation Analysis under Longitudinal Inclined Impact

**Figure 10:**
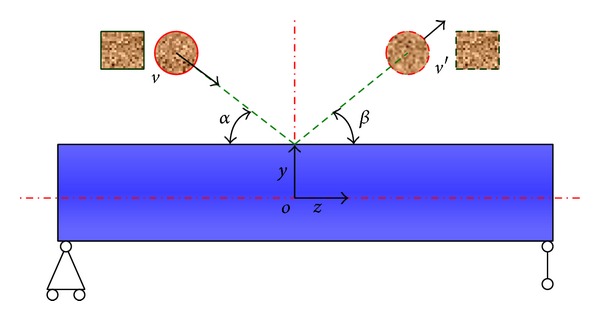
Schematic diagram of longitudinal inclined impact.

Rockfall may impact pipeline along different angles; therefore, the effect of impact angle on the pipeline deformation should be discussed. The schematic diagram of rockfalls impact pipeline under longitudinal inclined direction is shown in Figure 10. The incidence angle of the rockfall is *α*, the incidence velocity is *v*, the rebounding angle is *β*, the reflection velocity is *v*′, and the initial impact velocity is *v* = 60 m/s.

**Figure 11:**
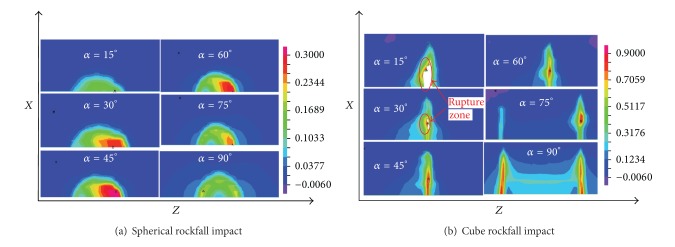
Plastic strain nephogram under longitudinal inclined impact.

The plastic strains of pipelines that were impacted by two kinds of rockfalls from different angles are shown in Figure 11.  Figure 11(a) shows that the plastic strain of impact zone is uneven when the impact angle is less than 90°. And the smaller the incidence angle, the more obvious this nonuniformity. When *α* = 90° (the radial impact mentioned above), the impact crater is a symmetric distribution on the *x*-axis. When *α* changes from 0° to 90°, the maximum plastic strain of the impact crater increases at first and then decreases, and it reaches the maximum value when *α* = 45°. As shown in Figure 11(b), when the incidence angle is smaller, only one cube edge of rockfall is in contact with the pipeline. As *α* increases gradually, the second edge gradually comes into contact with the pipeline. When *α* is 15° and 30°, the pipeline ruptures appear, and the smaller the incidence angle, the larger the rupture range. The largest plastic strain is the contact zone between rockfall edges and the pipeline.

**Figure 12:**
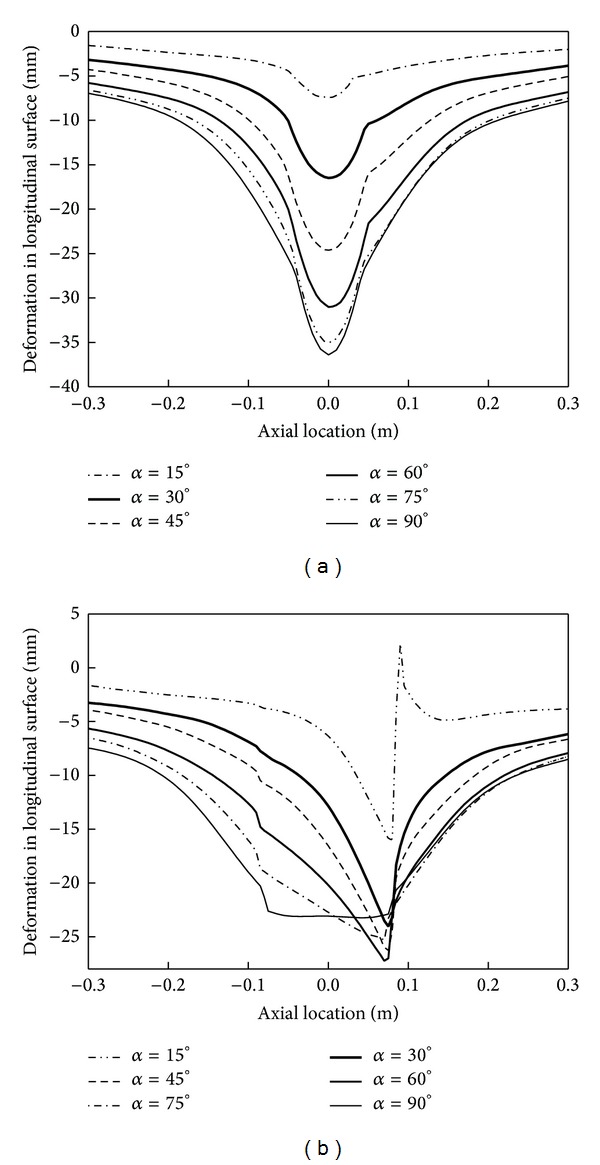
Pipeline deformation in longitudinal surface.

The deformations of pipeline in longitudinal surface under different incidence angles are shown in Figure 12.  Figure 12(a) shows that the smaller the incidence angle, the more inhomogeneous the deformation on both sides of the impact crater. The smaller *α*, the smaller the pipeline impact crater. As the increasing of *α*, the largest impact crater depth increases. In Figure 12(b), the impact crater caused by cube rockfall is a triangle when *α* ≤ 90°. When *α* = 15°, the mutation of the pipeline deformation curve appears, which means that the rupture occurred here. When *α* = 30°, the deformation curve of longitudinal cross section has no mutation, which indicates that the centre of the impact crater has no rupture.

### 4.3. Deformation Analysis under Transverse Inclined Impact

**Figure 13:**
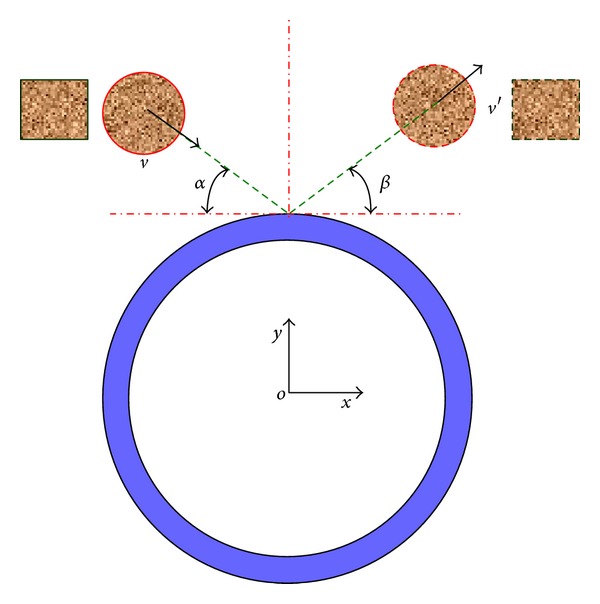
Schematic diagram of transverse inclined impact.

The schematic diagram of rockfalls impact pipeline under transverse inclined impact is shown in Figure 13. As the cube rockfall is not axisymmetric structure, this paper only considered the contact between the center of cube rockfall lower surface and the pipeline.

**Figure 14:**
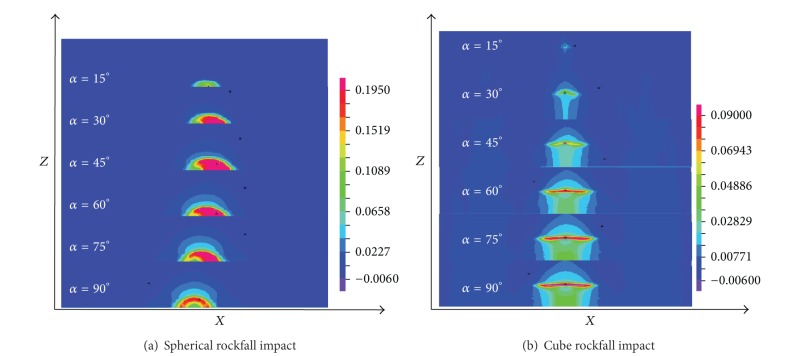
Plastic strain nephogram under transverse inclined impact.

The plastic strains of pipelines under different impact angles are shown in Figure 14.  Figure 14(a) shows that impact angle makes an uneven plastic strain distribution of impact crater. When *α* = 90°, the elliptical ring appears; the largest plastic strain zone is not in the center of the crater. As the incidence angle increases, the large plastic strain zone increases at first and then decreases. When *α* = 45°, the plastic strain reaches the maximum value. As shown in Figure 14(b), the whole plastic strain increases with the increasing of the incidence angle, and the large plastic strain zone also increases, which is mainly concentrated on the contact zone between cube edges and pipeline.

**Figure 15:**
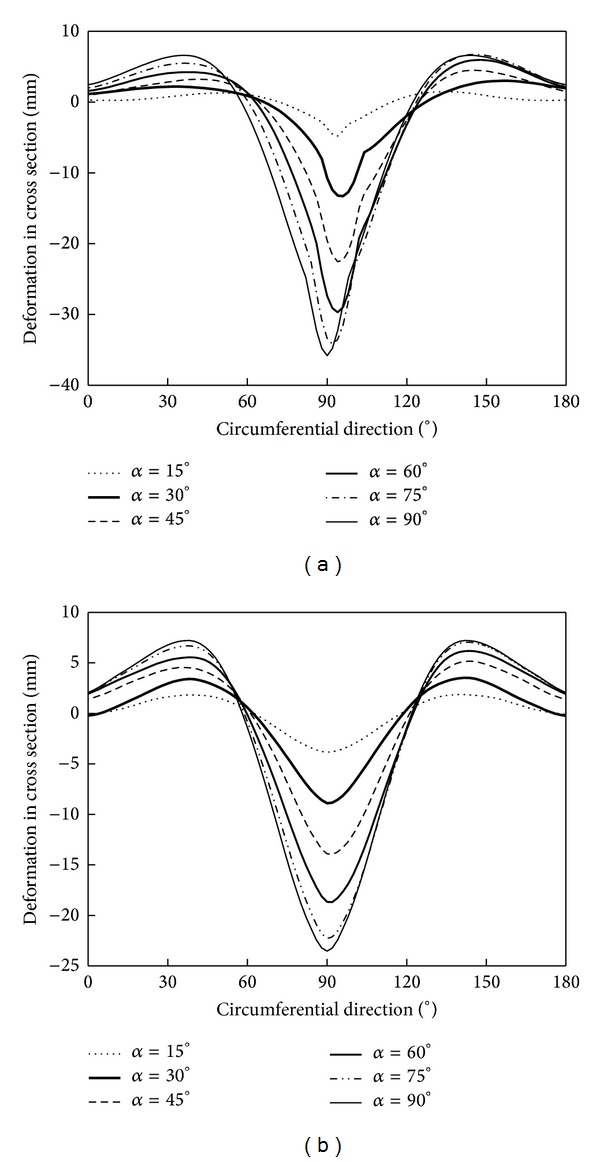
Pipeline deformation in cross surface.

Figure 15 shows the deformation curves of pipeline in cross section under different impact angles. The pipeline wall shows concave deformation along the circumferential direction 60° ~120°, while the wall shows outer concave deformation along the circumferential direction 60° ~120° and 120° ~180°. As *α* increases, the depth of the impact crater increases.  Figure 15(a) shows that the pipeline deformation along the 90° direction is not a symmetrical distribution. As shown in Figure 15(b), the problem of the cube rockfall impact pipeline can be considered as the collision contact problem between a plane and the pipeline.

### 4.4. Deformation Analysis under Lateral Eccentric Impact

**Figure 16:**
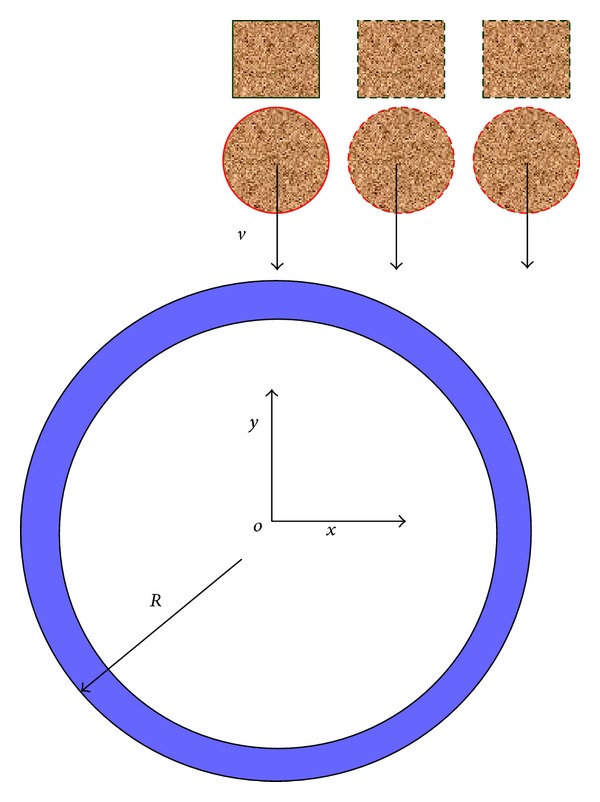
Schematic diagram of lateral eccentric impact.

Figure 16 shows the rockfalls impact pipeline from different lateral positions. In order to carry out quantitative description, it is defined as eccentricity *k* = *x*′/*R*, where *x*′ is the distance between rockfall center and pipeline center along the *x* direction and *R* is the outer radius of the pipeline.

**Figure 17:**
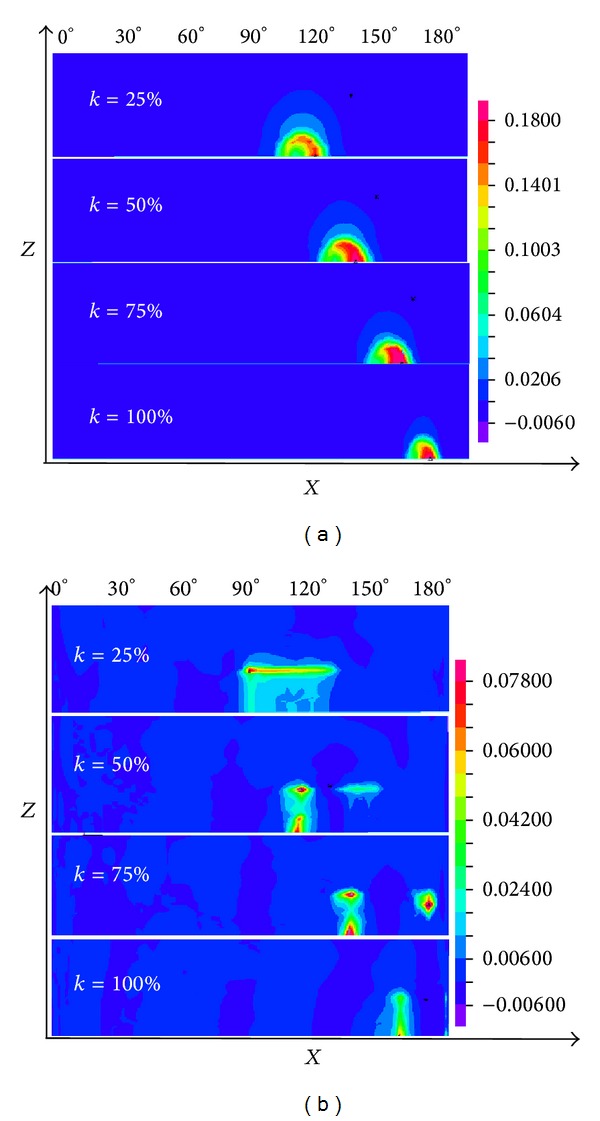
Plastic strain nephogram under lateral eccentric impact.

Plastic strains of pipelines impacted under different eccentricities are shown in Figure 17.  Figure 17(a) shows that pipelines' plastic strain zone gradually decreases with the increasing of the eccentricity *k*, and the plastic strain zone also gradually deviates from 90° center position with the increasing of *k*. As shown in Figure 17(b), the plastic strain zone of the pipeline also gradually decreases with the increasing of *k* and deviates from center position. The large plastic strain zone is mainly concentrated on the contact region of the cube edges and the pipeline.

**Figure 18:**
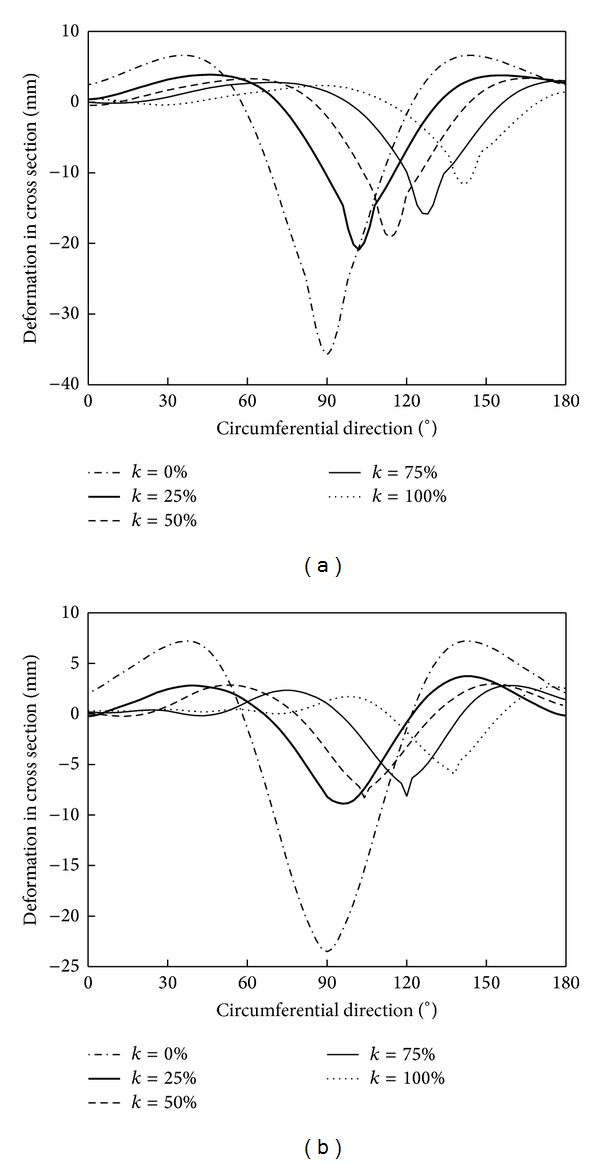
Pipeline deformation in cross section.

Figure 18 shows the deformation curves of the pipelines in the cross section under different eccentricities. With the increasing of *k*, the depth of the impact crater gradually decreases. And the change rate of impact crater depth caused by spherical rockfall is larger than cube rockfall.

## 5. Conclusions

The FE models of oil and gas pipelines impacted by spherical and cube rockfalls were established in this paper. The pipeline deformations under radial impact, longitudinal inclined impact, transverse inclined impact, and lateral eccentric impact were analyzed. The conclusions are summarized as follows.

1. Under radial impact, the crater impacted by spherical rockfall on the pipeline was deeper than by cube rockfall with the same volume.
2. When *α* changes from 0° to 90°, the maximum plastic strain of the pipeline caused by spherical rockfall increases at first and then decreases under longitudinal inclined impact. When *α* is 45°, the plastic strain reaches the maximum value. Specially, when *α* is small, cube rockfall can probably lead to pipeline's rupture.
3. The plastic strain distribution of pipeline is extremely uneven under longitudinal inclined impact. As *α* increases gradually, the plastic zone increases, and large plastic strain zone is mainly concentrated on contact zone between the cube rockfall edges and the pipeline.
4. Under lateral eccentric impact, the pipeline plastic strain zone decreases with the increasing of *k*. And the large plastic strain zone deviates from 90° center position with the increasing of *k*.
